# Effects of different medical masks on acoustic and aerodynamic voice assessment during the COVID-19 pandemic

**DOI:** 10.1097/MD.0000000000034470

**Published:** 2023-08-04

**Authors:** Mingwen Yu, Qianqian Jin, Weiming Zhang, Xin Sun, Yuxin Sun, Qing Xie

**Affiliations:** a Department of Rehabilitation Medicine, Ruijin Hospital, School of Medicine, Shanghai Jiao Tong University, Shanghai, China.

**Keywords:** acoustic voice analysis, aerodynamic analysis, COVID-19, ling WAVES, N95 mask, surgical mask

## Abstract

The purpose of the study was to investigate the effect of the surgical masks and N95 masks on the acoustic and aerodynamic parameters of voice assessment during the coronavirus disease 2019 pandemic. The challenge of the study was to enable each inexperienced participant to perform a number of acoustic and aerodynamic voice assessment in a qualified and homogeneous manner without and with medical masks, and to minimize the individual differences. There were 32 healthy participants recruited in the study, including 16 males and 16 females. The acoustic parameters analyzed included fundamental frequency, standard deviation of fundamental frequency (fundamental frequency standard deviation), percentage of jitter (%), percentage of shimmer (%), glottal-to-noise excitation ratio (GNE), and the parameters of irregularity, noise and overall severity. The aerodynamic parameters included s time, z time, s/z ratio and maximum phonation time. When wearing surgical masks, the GNE ratio (*P* = .043) significantly increased, whereas noise (*P* = .039) and s time (*P* = .018) significantly decreased. When wearing N95 masks, the percentage of shimmer (*P* = .049), s time (*P* = .037) and s/z ratio (*P* = .048) significantly decrease. In general, performing voice assessment with a medical mask proved to be reliable for most of the acoustic and aerodynamic parameters. It is worth noting that the shimmer (%), could be slightly impacted when wearing N95 masks. Wearing surgical masks might slightly influence the measurement of noise and higher GNE ratio. The s/z ratio could be affected when wearing N95 masks. The contribution of the study is to explore acoustic and aerodynamic parameters that might be easily affected by wearing masks during the voice assessment, and provide references for clinical evaluation of voice disorders during the pandemic of coronavirus disease 2019.

## 1. Introduction

In 2020, the World Health Organization declared a global pandemic caused by the severe acute respiratory syndrome coronavirus-2 that causes novel coronavirus disease (COVID-19).^[1]^ As a respiratory virus, the major transmission modes of severe acute respiratory syndrome coronavirus-2 virus are through contact, respiratory droplets and airborne.^[2]^ Wearing face masks can interrupt the dispersion of particles expelled through coughing or sneezing, preventing the transmission of respiratory diseases. If 2 people wear surgical masks, the rate of reduction in the risk of COVID-19 spreading was estimated to be at least 80%.^[3]^

Voice assessment is of great significance to the evaluation of voice quality, identifying voice disorders and assessing the prognosis of therapy. During the pandemic of COVID-19, performing voice analysis without a face mask could put both the patient and the physician at risk of infection. However, face masks can muffle speech sounds, especially higher frequencies which can help to differentiate similar sounds. It was reported that the acoustic effect of a speaker wearing a face mask was almost equivalent to the listener having a slight high-frequency hearing loss.^[4]^ In addition, the type of medical mask worn may uniquely affect the acoustic and speech perception, since mask types vary in their composition and the way they were designed to fit on the face.

The purpose of the study was to investigate the impact of wearing a surgical mask or N95 mask on healthy participants in terms of acoustic and aerodynamic parameters during the COVID-19 pandemic. We hypothesized that effects of different medical masks on voice quality would differ based on their unique filtering properties.

## 2. Methods

### 2.1. Participants and procedures

There were 32 healthy participants recruited in the study, including 16 males and 16 females. The mean age of the participants was 29.71 ± 5.12 (range 22 to 45) years old. The mean body mass index of the participants was 22.29 ± 3.12 kg/m^2^. All the participants received voice assessment in the Voice center at Ruijin Hospital, Shanghai Jiao Tong University School of Medicine from May 2022 to July 2022.

All participants were expected to meet the following inclusionary criteria: native speakers of Mandarin Chinese; between 18 and 45 years of age; and absence of comorbid health conditions affecting respiration and voice. The exclusionary criteria included: having recent voice problems or a voice disorder history; conditions that may affect normal voice function, including with history of smoking and chronic alcohol use; any respiratory infection within 2 weeks; any previous formal voice training or voice therapy; any laryngeal, throat or oral abnormality. The approval of ethics committee was obtained and informed consent was obtained from all of the participants prior to recruitment into our study.

A repeated-measures study was designed for the measurement of acoustics under different mask conditions. The mask conditions included without a mask, wearing a surgical mask, and an N95 mask. Participants were instructed to wear the medical masks to cover the nose up to the bridge, mouth and chin. In order to minimize the order effects, the mask conditions for evaluation were randomized. The subjective sensation of dyspnea was assessed using the modified Borg Scale (score of 0–10) each time before voice assessment.^[5]^

### 2.2. Aerodynamic and acoustic analysis

Participants were seated comfortably, and a WEVOSYS sound pressure level (SPL) meter microphone was positioned at a standard distance of 30 cm away from the oral cavity at an angle of 45 degrees. Recording was performed in a quiet room with ambient noise below 60dB SPL.

We used the ling WAVES software (Version 3.0; Wevosys, Forchheim, Germany) for the acoustic and aerodynamic analysis of voice samples. The ling WAVES software is a program used for voice and speech analysis, biological feedback, and documentation. The acoustic parameters analyzed included: fundamental frequency (F0), standard deviation of fundamental frequency (F0 SD), percentage of jitter (%), percentage of shimmer (%) and glottal-to-noise excitation ratio (GNE). The parameters of irregularity (roughness), noise (breathiness), and overall severity (hoarseness) were obtained from the middle 2-thirds portion of the sustained/a/voice samples.

The Voice Protocol module of ling WAVES can evaluate the aerodynamic parameters such as s time, z time, s/z ratio and maximum phonation time (MPT). The s/z ratio was collected by instructing the participants to take a deep breath and sustain each/s/ and/z/ sound as long as possible. Recovery time was provided after each maximum sustained sound. The longest/s/ and the longest/z/ were used to calculate the s/z ratio. The procedure for measuring MPT was as follows: participants took a deep breath and then produced the/a/ sound at a moderate pitch and volume as long as they could. Three measures were taken at 5-minute intervals, and the measure with the longest duration was noted. The entire procedure was performed by an experienced laryngologist.

The parameters used for dysphonia severity index (DSI) measurements included the highest fundamental frequency (F0-high in Hz), lowest intensity (I-low in dB), SPL, MPT, and jitter (%).

### 2.3. Statistical analysis

Statistical analysis was performed using the SPSS v16.0 software (SPSS, Inc., Chicago, IL). The continuous data were presented as the mean ± standard deviation. Paired *t* tests were performed to evaluate the change in vocal parameters before and after wearing medical masks. The differences in the normally distributed data among the study groups were analyzed using repeated-measures ANOVA test. The Friedman test was applied for a nonparametric comparison of the parameters according to the study groups. Two-sided *P* values < .05 were considered statistically significant.

## 3. Results

### 3.1. Comparison of the results of the acoustic and aerodynamic analysis of all 32 participants

The Borg Scale was significantly higher in participants wearing N95 masks than without masks and wearing surgical masks (*P* = .042). There showed no significant difference in Borg Scale when wearing surgical masks compared with without masks (*P* = .404).

The acoustic and aerodynamic parameters of the 32 participants are summarized in Table 1. When wearing surgical masks, the GNE ratio (*P* = .043) significantly increased, whereas noise (*P* = .039) and s time (*P* = .018) significantly decreased. When wearing N95 masks, the percentage of shimmer (*P* = .049), s time (*P* = .037) and s/z ratio (*P* = .048) significantly decreased. However, no statistically significant difference was observed in terms of the acoustic and aerodynamic parameters among the participants without masks, with surgical masks and with N95 masks.

**Table 1:** Comparison of the results of the acoustic and aerodynamic analysis according to the groups.

	No mask	Surgical mask	N95 mask	*P* value† (No mask vs Surgical mask)	*P* value† (No mask vs N95 mask)	*P* value‡ (No mask vs Surgical mask vs N95 mask)
F0 (Hz)	213.04 ± 64.65	209.67 ± 56.16	214.59 ± 56.74	.575	.798	.944
F0 SD (Hz)	0.619 ± 0.323	0.833 ± 0.626	0.658 ± 0.367	.064	.563	.144
Jitter (%)	0.0875 ± 0.0583	0.0788 ± 0.0364	0.0859 ± 0.0323	.403	.878	.697
Shimmer (%)	6.927 ± 2.386	6.460 ± 2.076	6.189 ± 2.085	.304	.049*	.397
GNE ratio	0.8269 ± 0.0817	0.8606 ± 0.0676	0.8450 ± 0.0764	.043*	.167	.207
Irregularity	0.8116 ± 0.1117	0.7956 ± 0.1181	0.7959 ± 0.0954	.448	.508	.800
Noise	0.3791 ± 0.1329	0.3228 ± 0.1095	0.3500 ± 0.1267	.039*	.172	.195
Overall severity	2.98 ± 11.683	0.865 ± 0.1196	0.8787 ± 0.1009	.315	.318	.357
MPT(s)	24.271 ± 7.732	24.414 ± 9.521	22.552 ± 6.624	.908	.098	.590
DSI	5.997 ± 1.945	6.15 ± 2.035	6.047 ± 1.951	.589	.819	.951
S time (s)	18.8191 ± 8.8942	16.6134 ± 6.9616	16.1522 ± 7.4507	.018*	.037*	.349
Z time (s)	22.3925 ± 7.8436	21.230 ± 7.6996	21.6656 ± 7.5920	.198	.511	.831
s/z ratio	0.8391 ± 0.2030	0.8212 ± 0.2781	0.7662 ± 0.2174	.691	.048*	.438

DSI = dysphonia severity index, F0 = fundamental frequency, F0-Hz = highest fundamental frequency, GNE = glottal-to-noise excitation, MPT = maximum phonation time, SD = standard deviation.

* *P* < .05.

† Paired *t* test.

‡ One-way ANOVA test.

### 3.2. Comparison of the results of the acoustic and aerodynamic analysis of male and female participants

Since the gender of the participants may affect the parameters in voice analysis, the data for male and female participants were evaluated separately. The Acoustic and aerodynamic parameters of male and female participants are summarized in Table 2 and Table 3, respectively. There were no statistically significant differences in terms of acoustic and aerodynamic parameters among male participants. Among female participants, the s time was significantly decreased when wearing N95 masks (*P* = .043).

**Table 2:** Comparison of the results of the acoustic and aerodynamic analysis according to the groups of female participants.

	No mask	Surgical mask	N95 mask	*P* value† (No mask vs Surgical mask)	*P* value† (No mask vs N95 mask)	*P* value‡ (No mask vs Surgical mask vs N95 mask)
F0 (Hz)	266.52 ± 37.43	256.19 ± 37.91	262.24 ± 32.77	.444	.733	.720
F0 SD (Hz)	0.717 ± 0.318	1.124 ± 0.751	0.801 ± 0.367	.059	.492	.073
Jitter (%)	0.0950 ± 0.0768	0.0725 ± 0.0399	0.0969 ± 0.0342	.307	.929	.369
Shimmer (%)	6.772 ± 2.832	5.877 ± 1.970	5.656 ± 1.734	.307	.189	.334
GNE ratio	0.8131 ± 0.0913	0.8469 ± 0.747	0.8456 ± 0.804	.261	.294	.429
Irregularity	0.7994 ± 0.1229	0.7538 ± 0.1144	0.7919 ± 0.0775	.286	.838	.438
Noise	0.4025 ± 0.1486	0.3469 ± 0.1220	0.3475 ± 0.1322	.256	.277	.414
Overall severity	5.05 ± 16.52	0.84 ± 0.126	0.87 ± 0.098	.324	.320	.364
MPT (s)	21.232 ± 6.373	21.786 ± 7.514	20.536 ± 6.101	.824	.754	.870
DSI	5.769 ± 1.644	6.112 ± 2.221	6.150 ± 1.782	.622	.534	.823
S time (s)	21.8425 ± 9.8660	17.1894 ± 6.8297	15.9269 ± 5.3337	.131	.043*	.078
Z time (s)	23.8350 ± 7.8310	21.7950 ± 8.9767	20.9463 ± 6.2264	.499	.257	.561
s/z ratio	0.9025 ± 1.8041	0.8556 ± 0.3028	0.7881 ± 0.1929	.601	.096	.385

DSI = dysphonia severity index, F0 = fundamental frequency, F0-Hz = highest fundamental frequency, GNE = glottal-to-noise excitation, MPT = maximum phonation time, SD = Standard deviation.

* *P* < .05.

† Paired *t* test.

‡ One-way ANOVA test.

**Table 3:** Comparison of the results of the acoustic and aerodynamic analysis according to the groups of male participants.

	No mask	Surgical mask	N95 mask	*P* value† (No mask vs Surgical mask)	*P* value† (No mask vs N95 mask)	*P* value‡ (No mask vs Surgical mask vs N95 mask)
F0 (Hz)	159.57 ± 33.69	163.15 ± 21.56	166.94 ± 27.14	.722	.501	.758
F0 SD (Hz)	0.522 ± 0.307	0.543 ± 0.254	0.515 ± 0.316	.837	.951	.963
Jitter (%)	0.0800 ± 0.0318	0.0850 ± 0.0327	0.0750 ± 0.0271	.664	.636	.655
Shimmer (%)	7.081 ± 1.923	7.044 ± 2.073	6.722 ± 2.317	.958	.637	.870
GNE ratio	0.8406 ± 0.0712	0.8744 ± 0.0588	0.8438 ± 0.0748	.154	.904	.316
Irregularity	0.8238 ± 0.1018	0.8735 ± 0.1096	0.8000 ± 0.1131	.716	.537	.615
Noise	0.3556 ± 0.1152	0.2987 ± 0.0932	0.3525 ± 0.1252	.135	.942	.281
Overall severity	0.9062 ± 0.8461	0.8925 ± 0.1100	0.8856 ± 0.1065	.695	.549	.842
MPT (s)	27.309 ± 7.952	27.041 ± 10.777	24.568 ± 6.692	.937	.300	.671
DSI	6.220 ± 2.237	6.290 ± 1.903	5.940 ± 2.161	.960	.720	.919
S time (s)	15.7956 ± 6.8299	16.0375 ± 7.2667	16.3775 ± 9.2828	.923	.841	.978
Z time (s)	20.9500 ± 7.8348	20.6644 ± 6.4231	22.3850 ± 8.9021	.911	.632	.800
s/z ratio	0.7756 ± 0.2067	0.7869 ± 0.2562	0.7444 ± 0.2439	.892	.699	.871

DSI = dysphonia severity index, F0 = fundamental frequency, F0 SD = standard deviation of fundamental frequency, F0-Hz = highest fundamental frequency, GNE = glottal-to-noise excitation, MPT = maximum phonation time, SD = standard deviation.

* *P* < .05.

† Paired *t* test.

‡ One-way ANOVA test.

## 4. Discussion

Voice assessment is an objective, noninvasive and easy-to-apply method to evaluate voice quality. During the pandemic, performing voice analysis without masks could increase the risk of COVID-19 transmission, putting the patients and the voice therapist at risk. It is estimated that the reduction rate in the risk of spreading COVID-19 between 2 people is at least 80% if they wear surgical masks.^[3]^ Therefore, patients are required to wear medical masks when voice analysis is conducted. However, it cannot be ignored that people who wear medical masks may experience vocal fatigue, poor speech intelligibility and poor coordination between speech and breathing.^[6]^ In our study, a significantly higher Borg Scale was observed in participants when wearing N95 masks rather than wearing surgical masks, which suggested that N95 masks may have a certain influence on the accuracy of vocal assessment.

The study of Goldin et al^[7]^ indicated that wearing any type of mask could cause a low-pass filter effect, impairing the highest frequencies (2000–7000Hz) of the voice. The decibel reduction ranged from 3 to 4dB for surgical masks to 12dB for N95 masks. Some recent studies have investigated the potential effects of medical masks on several basic parameters.

The F0 is the intrinsic frequency of vocal fold vibration. The value of F0 is tightly associated with the length, tension and mass of vocal folds and the subglottic pressure.^[8]^ Previous studies reported that F0 can be impacted by several factors, including age, ethnological background and vocal fold length. Perturbation is used to describe the irregularity of vocal fold vibration, mainly including jitter (%) and shimmer (%).^[9]^ Jitter (%), also known as frequency perturbation, measures the variation from the cycle-to-cycle within the fundamental frequency of a voice signal. Shimmer (%), known as amplitude perturbation, measures the variation from the cycle-to-cycle within the amplitude of voice.^[10]^ The smaller these 2 parameters are, the more stable the vocal fold vibration is.

In the studies of Cavallaro et al^[11]^ and Fiorella et al,^[12]^ they found no significant difference in F0, shimmer and jitter values without and with surgical masks. Nevertheless, results of the study of Lin et al^[13]^ showed a significantly decrease in both jitter and shimmer when wearing medical masks, with an insignificantly increasing trend of F0. Previous studies indicated that the loudness could influence the jitter (%) and shimmer (%), to a certain extent.^[14]^ The value of jitter (%) and shimmer (%) may decrease when the volume increases. In our study, only shimmer (%) measured significantly decreased when the participants wore N95 masks, but not surgical masks. However, F0 was not detect to increase when wearing masks. Considering medical masks could act as a voice filter,^[15]^ the measurement of shimmer (%) may be affected by masks to some degree.

F0 SD refers to the F0 variation that is captured by measuring the standard deviation in voice pitch. F0 SD was considered as a more reliable index than jitter and shimmer for the assessment of voice quality, since a rapidly shifting F0 may not alter the perturbation parameters.^[16]^ In the current study, the value of F0 SD was not impacted while wearing masks. We believe that F0 SD is still a reliable and sensitive vocal analysis index for vocal analysis with a medical mask.

The ling WAVES software (https://www.wevosys.com/products/lingwaves/lingwaves) we applied included a number of objective parameters such as irregularity, noise and overall severity, which assesses roughness, breathiness and the general hoarseness level of voice, respectively. Through the Vospector module of lingWAVES, this software enables perceptual evaluation of voice quality.^[17]^ In the present study, participants wearing surgical masks were observed significantly lower values of noise. Since wearing masks could impact the breathiness, the accuracy of the measurement of the parameter of noise could be affected to some extent.

GNE measures the degree of voice turbulence by applying the method of inverse filtering of vocal signal.^[18]^ GNE is considered as a reliable voice evaluation measure since it is better at discriminating between healthy and deviated voices.^[19]^ The value of GNE lower than the reference value of 0.5% may be considered deviated. GNE represents an interesting approach to quantifying the amount of excitation caused by vocal fold oscillations versus the excitation owing to turbulent noise. Hence, GNE is closely related to breathiness. In the present study, the GNE value of participants became significantly higher when wearing surgical masks, which can partly be explained by the decrease in breathiness.

The MPT referred to the longest period during the sustained phonation of a vowel sound. MPT is the easiest and most commonly used aerodynamic parameter, which can reflect the adjustment function of the larynx, the ability of phonation, vital capacity and general health status.^[20]^ In the study of Fiorella et al^[12]^ and Gojayev et al,^[21]^ there showed no statistically significant difference in terms of MPT when with surgical masks and without surgical masks. In the study of Lin et al,^[13]^ MPT in the participants above 45 years old was influenced more than that in below 45 years old. They assumed that medical masks acted as a physical barrier and decreased the vital capacity of healthy subjects. The inability of the aged people to compensate for the loss of vital capacity makes the MPT shorter. In our study, most of the participants were below 45 years old. The measurement of MPT was not impacted when wearing medical masks.

DSI is a linear combination of several parameters of voice, which can be obtained from basic acoustic and aerodynamic analysis.^[22]^ It was reported that DSI can be helpful in describing differences in vocal capability and distinguishing disordered from normal subjects. According to our knowledge, no other studies to date have investigated the effect of wearing medical masks on DSI. Our results indicated that wearing medical masks would not influence the assessment of DSI in vocally healthy participants.

The s/z ratio measures the differential duration which has been recommended for voice evaluations and diagnosis. Participants with normal vocal folds are expected to prolong the duration of voiceless/s/ and voiced/z/ phonemes for about the same, resulting in the s/z ratio of approximating 1. It is hypothesized that patients with laryngeal diseases would unable to prolong the/z/ for the same duration as/s/, because of the decrease in glottal resistance and the increase of glottal airflow, which caused a shortened phonation time. As for vocally healthy participants, there are also reasons to assume that/z/ should be prolonged for a greater duration than/s/, because of the increased glottal efficiency and decreased airflow.^[23]^ In the study of Gojayev et al,^[21]^ no significant difference was found in s/z ratio in vocal analysis performed without masks, surgical masks and FFP3 respirators. In our study, however, wearing surgical masks or N95 masks could weaken the measurement of the voiceless/s/ rather than the voiced/z/. In addition, wearing N95 masks could impact the measurement of s/z ratio. We assumed that participants with underlying abnormalities of voice could be missed when wearing N95 masks during the voice evaluation.

Since gender is the most important factor affecting vocal parameters, it is necessary to investigate the parameters separately according to gender. In the study of Lin et al, no significant difference was observed in terms of F0, jitter (%), shimmer (%) and MPT between with and without medical masks among male and female participants, which demonstrated the impact of medical masks was similar regardless of gender. In the study of Gojayev et al,^[21]^ among female participants, no significant differences were observed in terms of F0, jitter (%), shimmer (%), MPT and s/z ratio when without masks, with surgical masks or FFP3 masks. As for male participants, there showed no significant difference in terms of F0 and s/z ratio in all 3 conditions. Jitter (%) was significantly lower when wearing surgical masks, and MPT was measured lower when wearing surgical masks and FFP3 masks. In our study, only s time was observed to be decreased when wearing N95 masks in female participants, which suggested that the measurement of s/z ratio in women was more likely to be affected when wearing N95 masks.

There were several limitations in our study. First, the sample was relatively small. Moreover, all participants involved in our study were physically health and under 45 years of age. Since voice assessment is usually performed in patients with voice disorders, and the majority of whom is elder people, our results might not reflect the actual impact of medical masks during the voice assessment. Therefore, we will enlarge the sample size and further group participants by different ages in the future. Further studies should be conducted focusing on the real impact of different masks in patients with voice disorders.

## 5. Conclusion

In general, performing voice assessment with a medical mask was proved to be reliable for most of the acoustic and aerodynamic parameters. The impact of medical masks on gender was not obvious. It is worth noting that the perturbation parameter, shimmer (%), could be slightly impacted when wearing N95 masks. Wearing surgical masks might influence the breathiness, which leads to a significantly lower value of noise and a higher GNE ratio. The measurement of s/z ratio, an aerodynamic parameter, could be slightly affected when wearing N95 masks. According to our results, physicians can personalize the choice of subjects to wear a surgical mask or N95 mask for voice assessment based on the vocal parameters required.

## Acknowledgments

Special thanks to the citizen science volunteers for their collaboration and involvement in this project.

## Author contributions

**Conceptualization:** Mingwen Yu, Qing Xie.

**Data curation:** Mingwen Yu, Qianqian Jin, Weiming Zhang.

**Formal analysis:** Mingwen Yu, Qianqian Jin.

**Funding acquisition:** Qing Xie.

**Investigation:** Qianqian Jin.

**Methodology:** Qianqian Jin.

**Resources:** Xin Sun.

**Software:** Xin Sun, Yuxin Sun.

**Supervision:** Xin Sun, Qing Xie.

**Validation:** Xin Sun, Yuxin Sun.

**Writing – original draft:** Mingwen Yu, Weiming Zhang.

**Writing – review & editing:** Weiming Zhang, Qing Xie.
